# Uniaxially fixed mechanical boundary condition elicits cellular alignment in collagen matrix with induction of osteogenesis

**DOI:** 10.1038/s41598-021-88505-z

**Published:** 2021-04-27

**Authors:** Jeonghyun Kim, Keiichi Ishikawa, Junko Sunaga, Taiji Adachi

**Affiliations:** 1 Institute for Frontier Life and Medical Sciences, Kyoto University, Kyoto, 606-8507 Japan; 2 Department of Micro Engineering, Graduate School of Engineering, Kyoto University, Kyoto, 606-8507 Japan

**Keywords:** Biomaterials - cells, Biotechnology, Biomedical engineering

## Abstract

Osteocytes differentiated from osteoblasts play significant roles as mechanosensors in modulating the bone remodeling process. While the well-aligned osteocyte network along the trabeculae with slender cell processes perpendicular to the trabeculae surface is known to facilitate the sensing of mechanical stimuli by cells and the intracellular communication in the bone matrix, the mechanisms underlying osteocyte network formation remains unclear. Here, we developed a novel in vitro collagen matrix system exerting a uniaxially-fixed mechanical boundary condition on which mouse osteoblast-like MC3T3-E1 cells were subcultured, evoking cellular alignment along the uniaxial boundary condition. Using a myosin II inhibitor, blebbistatin, we showed that the intracellular tension via contraction of actin fibers contributed to the cellular alignment under the influence of isometric matrix condition along the uniaxially-fixed mechanical boundary condition. Furthermore, the cells actively migrated inside the collagen matrix and promoted the expression of osteoblast and osteocyte genes with their orientations aligned along the uniaxially-fixed boundary condition. Collectively, our results suggest that the intracellular tension of osteoblasts under a uniaxially-fixed mechanical boundary condition is one of the factors that determines the osteocyte alignment inside the bone matrix.

## Introduction

The bone constantly adapts to the dynamic environment by changing its structure, referred to as bone remodeling. Osteocytes are the most abundant cells in the bone and perform a unique function as the commander in sensing changes in the mechanical environment^1,2^. They are also known to control remodeling activities, including bone resorption by osteoclasts and bone formation by osteoblasts^3,4^. Osteoblasts produce type I collagen fibers to form the osteoid matrix, and further mineralize the matrix by secreting matrix vesicles that deposit calcium phosphate crystals within the collagen fibers^5^. During bone formation, a certain part of osteoblasts is known to become captured in the osteoid matrix, terminally differentiating into mechano-sensing osteocytes.

Bone remodeling has been shown to occur more actively in the trabeculae of the cancellous bone than in the dense cortical bone^6^. During the initial stage of bone formation, known as the modeling phase, osteoblasts produce an immature bone structure termed primary trabeculae, while secondary trabeculae are formed during the remodeling phase. It is also known that the arrangement and morphology of osteocytes inside the bone matrix are altered in the secondary trabeculae^7^. Whereas the osteocytes are randomly distributed in the primary trabeculae, those in the secondary trabeculae are well-distributed along the long axis of the trabeculae^8,9^. Moreover, the dendritic processes of the osteocytes have been shown to be perpendicularly elongated to the surface of the trabeculae. The arrangement of osteocytes is believed to be advantageous for the cells with regard to sensing mechanical stimuli and communication with other cells on the trabecular surface. In order to investigate the mechanisms underlying the formation of the organized arrangement of the osteocytes with the aligned processes, an appropriate in vitro experimental model is required, especially to induce cellular arrangements similar to that in the bone matrix.

In order to reveal the mechanism of the cellular alignments of osteoblasts and osteocytes in the bone matrix, we introduced a three-dimensional (3D) in vitro culture system using a collagen matrix. While several studies have utilized an in vitro collagen-based model for analyzing the process of osteogenesis differentiation^10–14^, this is the first study to attempt the modulation of the cellular alignment in the collagen matrix. Notably, we developed a novel method to exert a uniaxially-fixed mechanical boundary condition in our collagen culture system. Using this collagen-based system, we evoked cellular alignment and migration of osteoblast-like cells in the collagen matrix with the induction of osteogenesis and shed light on the mechanism of the formation of the aligned osteocytes in the bone matrix.

## Results

### Intracellular tension of osteoblast-like cells under uniaxially-fixed mechanical boundary condition induced cell alignment on the surface of collagen matrix after 1 day

**Figure 1:**
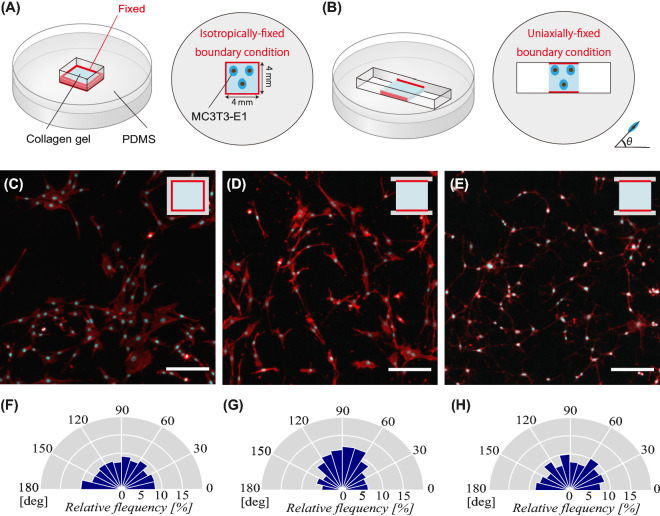
Schematic illustration of 3D collagen in vitro systems, (**A**) all-side fixed matrix and (**B**) 2-side fixed matrix, used to exert the isotropically- and uniaxially-fixed mechanical boundary condition, respectively. Images of DAPI and ACTIN staining of osteoblast-like cells cultured on (**C**) all-side fixed matrix and (**D**) 2-side fixed matrix for 1 day. (**E**) Images of DAPI and ACTIN staining of cells cultured on the 2-side fixed matrix for 1 day in the presence of blebbistatin. The ratios of cellular alignment of (**C**), (**D**), and (**E**) are depicted in (**F**), (**G**), and (**H**), respectively. Scale bars = 100 μm.

In this experiment, we fabricated a square-shaped in vitro system using a collagen matrix, which enabled the application of a fixed mechanical boundary condition to the borders of the collagen matrix contacting PDMS. Red lines around the collagen matrix depicted in Fig. 1A and B indicate the fixed mechanical boundary condition to the PDMS borders. As shown in Fig. 1A, all four sides of the collagen matrix were fixed with PDMS, providing an isotropically-fixed mechanical boundary condition (defined as the all-side fixed matrix). On the contrary, the 2-side fixed borders, as well as the other 2-side free borders shown in Fig. 1B, enabled the induction of the uniaxially-fixed mechanical boundary condition along a direction between the two fixed sides (defined as the 2-side fixed matrix).

We subcultured osteoblast-like MC3T3-E1 cells for 1 d in these two different collagen systems. Figure 1C and F show that cells cultured on the all-side fixed matrix were randomly distributed, with 33.2% of them being arranged between 60 and 120 degrees (Rayleigh’s test, *p* ≥ 0.05). On the other hand, cells subcultured on the 2-side fixed matrix were observed to be more aligned in a direction along the axis of the uniaxial mechanical boundary condition, as indicated in Fig. 1D and G, resulting in 45.3% of cells oriented between 60 and 120 degrees (Rayleigh’s test, *p* < 0.05).

Figure 1E and H show cells cultured in the 2-side fixed matrix for 1 d in the presence of blebbistatin, which was utilized as a myosin II inhibitor. Blebbistatin was added to investigate whether the intracellular tension in the collagen matrix was involved in the cellular alignment under the uniaxially-fixed mechanical boundary condition. Accordingly, we observed that the cell alignment distribution between 60 and 120 degrees became 32.6% (Rayleigh’s test, *p* ≥ 0.05), as shown in Fig. 1H, indicating that cellular alignment was randomly distributed similar to that in the all-side fixed matrix depicted in Fig. 1F.

In order to quantify the cellular alignment, the order parameter of cellular alignment *A* was utilized; $A=-<\mathrm{cos}2\theta >$. When the value of cellular alignment *A* became 0, it indicated a random cellular orientation. On the other hand, a value of 1 and -1 for *A* indicated that the cellular alignment was parallel and perpendicular to the axis of the uniaxial mechanical boundary condition, respectively, as shown in Fig. S1. The mean ± standard error of cellular alignment *A* for all-side fixed matrix and 2-side fixed matrix in the absence of blebbistatin, and 2-side fixed matrix in the presence of blebbistatin were − 0.014 ± 0.136, 0.194 ± 0.152, and 0.018 ± 0.053, respectively. These results indicated that cellular alignment in the all-side fixed matrix and 2-side fixed matrix in the presence of blebbistatin were randomly distributed, whereas that in the 2-side fixed matrix in the absence of blebbistatin was significantly oriented in parallel with the uniaxial boundary condition (*p* < 0.05). The addition of blebbistatin diminished the distributed cellular alignment and reversed to a random distribution in the 2-side fixed matrix (*p* < 0.01).

### Migrated cells inside the collagen matrix aligned along the uniaxially-fixed mechanical boundary condition

**Figure 2:**
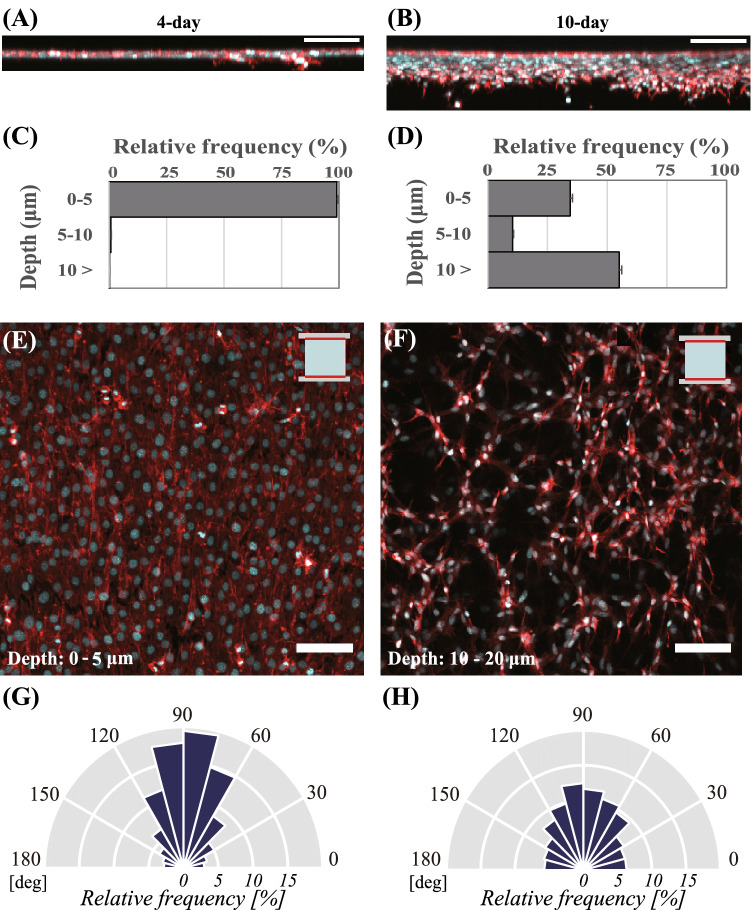
Image of DAPI and ACTIN staining in the vertical-section of cells subcultured in the 2-side fixed matrix after (**A**) 4-days and (**B**) 10-days incubation. Migrated cellular populations of osteoblast-like cells depending on the depth of the 2-side fixed collagen matrix at 0–5, 5–10, and 10 µm or deeper after (**C**) 4-days and (**D**) 10-days incubation. Bars represent the mean ± standard error. Image of DAPI and ACTIN staining depicting the cellular morphology (**E**) on the surface of the matrix at 0–5 µm depth after 4 days incubation and (**F**) at 10–20 µm depth inside the matrix after 10 days incubation. Ratio of cellular alignment (**G**) on the surface of the matrix after 4 days incubation and (**H**) at 10–20 µm depth in the matrix after 10 days incubation. Scale bars = 100 μm.

We then investigated the migration of osteoblast-like cells and their cellular alignments in the 2-side fixed collagen matrix for a longer period of time (4 days and 10 days). After 4 days of cultivation, cells were observed to become confluent on the surface of the collagen gel, as shown in Fig. 2A, C, and E. In Fig. 2A and C, as only 0.2% of cells were shown to migrate to 10 µm or deeper, most of the cells cultivated for 4 d were noted to stay on the surface of the collagen matrix (0–5 µm). The uniaxial mechanical boundary condition exerted by the 2-side fixed collagen matrix was also demonstrated to induce cell alignment along the boundary condition, causing 63.5% of cells to be orientated between 60 and 120 degrees on the surface of the matrix, as depicted in Fig. 2E and G. On the other hand, after 10 d of cultivation, whereas 35.6% of cells were observed on the surface of the collagen matrix, 55.1% of cells were observed to migrate inside the collagen at 10 µm or deeper, as shown in Fig. 2B and D. Particularly, the rate of cell alignment between 60 and 120 degrees at 10 and 20 µm depth was 44.5%, as depicted in Fig. 2F and H. These results implied that the collagen matrix allowed the cultured cells to migrate inside the matrix after 10 days. Moreover, these findings suggested that the uniaxially-fixed mechanical boundary condition evoked cellular alignment in the migrated cells.

With regard to cellular migration after 10 days incubation, we also reported the heterogeneity in the migratory behaviors shown in Fig. S2A and Movie S1. Particularly, in Fig. S2B, groups of cells in areas where the cell density was high (yellow box) were shown to migrate inside the matrix, whereas cells at low cell density (green box) stayed on the surface of the matrix. Notably, cells at the localized area were observed to migrate as a group and not as single entities. Hence, osteoblast-like cells in the collagen matrix exhibited heterogeneity in their cell migratory behavior.

### In vitro collagen model with boundary condition induced osteogenesis within 10 days

**Figure 3:**
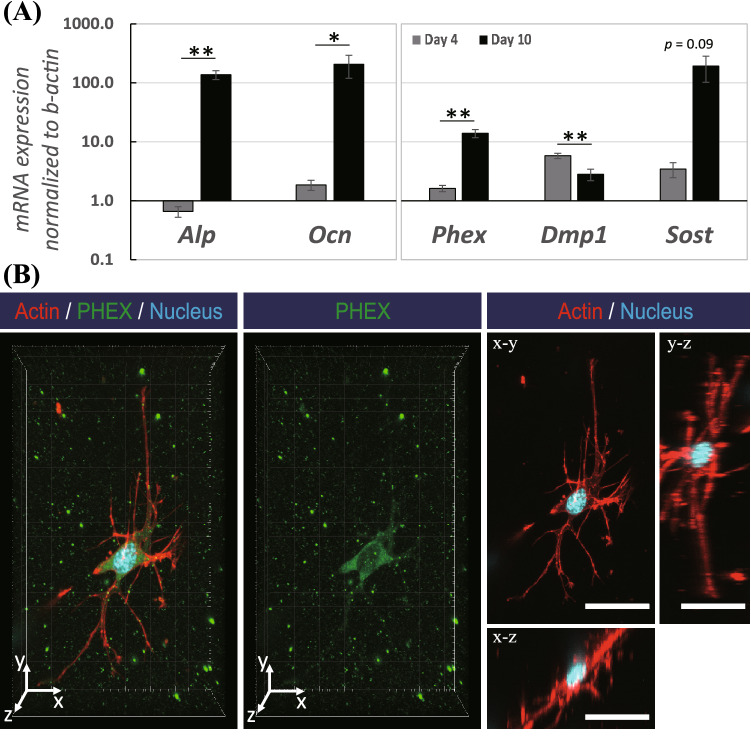
(**A**) The mRNA expressions of genes in osteoblast-like cells in the 2-side fixed matrix after 4 d and 10 d incubation. The mRNA expression of *Alp*, *Ocn*, *Phex*, *Dmp1*, and *Sost* was measured by real-time PCR and normalized to that of *β-actin*. Bars represent the mean ± standard error (*n* = 8; *p*-value was calculated by Student’s *t*-test; *p < 0.05, **p < 0.005). (**B**) PHEX (green) immunostaining counterstained with actin rhodamine-phalloidin (red) and DAPI (cyan) of a cell migrated at 10–20 µm depth in the 2-side fixed matrix. Scale bars = 30 μm.

In order to examine the level of gene expression for osteogenesis markers in the osteoblast-like cells subcultured in the collagen gel at day 4 and day 10, we conducted real-time-PCR (RT-PCR), as shown in Fig. 3A. Compared with day 4, osteoblast markers were observed to be significantly and highly up-regulated at day 10; *Alp* (257-fold change; *p* < 0.005) and *Ocn* (132-fold change; *p* < 0.05). The longer term of cultivation in the collagen matrix was also demonstrated to result in altered osteocyte markers, such as *Phex* (9.27-fold change; *p* < 0.005), *Dmp1* (0.48-fold change; *p* < 0.005), and *Sost* (60-fold change; *p* = 0.09). In order to examine the expression of an osteocyte marker, we carried out PHEX immunostaining for the cells migrated inside the collagen matrix after 10 d of cultivation, as shown in Fig. 3B. As a result of PHEX (green) immunostaining counterstained with rhodamine-phalloidin for actin filaments (red), we observed that the migrated cells expressed the PHEX marker inside the cell body. Particularly, the cells with PHEX expression were noted to retain a specific morphology resembling that of osteocytes with many slender cytoplasmic processes.

## Discussion

In bone remodeling, a part of osteoblasts is known to be captured in the bone matrix and terminally differentiated into osteocytes, followed by the mineralizing process. While the osteocytes are reported to be aligned in the direction along the longitudinal axis of the bone with cellular processes parallel to the surface of the trabecular bone^7^, the key factors or mechanisms of this cellular alignment in the bone matrix remain unknown. This alignment of osteocytes is thought to be advantageous for osteocytes, enabling them to sense mechanical stimuli with higher sensitivity and communicate the cells on the trabecular surface^15^. In order to achieve a better understanding of the alignment of osteoblasts and osteocytes in the bone matrix, we established a 3D in vitro experimental model using a collagen matrix exerting a uniaxially-fixed mechanical boundary condition, which mimics the local mechanical environments in the osteoid matrix similar to a remodeling packet on a single trabecula. Type I collagen is known to be the main constituent of the osteoid matrix and has been often utilized for in vitro bone formation or osteogenic differentiation studies^11,12,16^. The novelty of this study was the introduction of a uniaxially-fixed mechanical boundary condition in the collagen matrix, which successfully induced the cellular alignment on and inside the matrix. As represented in Fig. 1, cells under the isotropic boundary condition in the all-side fixed matrix were randomly distributed, whereas the 2-side fixed matrix evoked the cellular alignment along the uniaxial boundary condition. Hence, our novel model exploiting the uniaxially-fixed boundary condition successfully induced cellular alignment, indicating the relationship to the intercellular mechanical condition.

**Figure 4:**
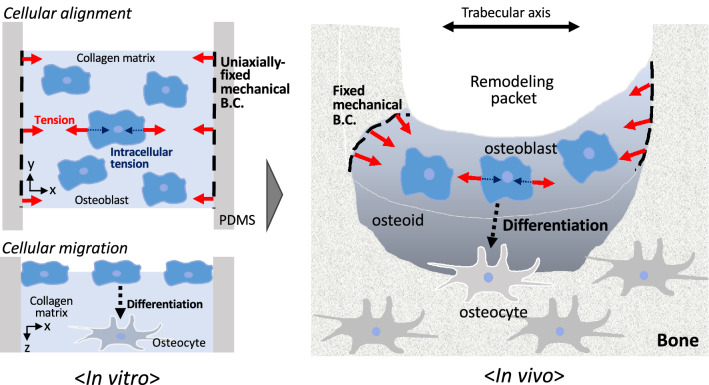
Schematic diagram illustrating the significance of intracellular tension and uniaxially-fixed boundary condition in the bone matrix to understand the formation process of in vivo osteocyte network.

In the present study, we assumed that the intracellular tension of osteoblast-like cells balanced with the intercellular tension might play a significant role in their cellular alignment. The intracellular tension induced by subcultured cells on the collagen matrix brought about stabilization and polymerization of cytoskeletal actin filaments along the boundary condition, further enhancing the intracellular tension. This positive mechano-feedback eventually strengthened the intracellular tension. Whereas the actin filaments are known to generate the intracellular tension by binding with motor proteins such as myosin II, the polymerization of actin is known to be facilitated by mechanical forces^17,18^. Here, in order to examine the involvement of contractile actin fibers in cellular alignment induced by the intracellular tension, we utilized blebbistatin as a myosin II inhibitor. The addition of blebbistatin reduced the extent of cellular alignment even in the 2-side fixed matrix. Previous studies reported that cells were capable of sensing and adapting against the mechanical force via modulation of the intracellular tension^19–21^, so that cells gradually changed their morphology and orientation. Our study revealed that the sensing capability of cells in response to the mechanical boundary condition was inhibited by the addition of blebbistatin, implying the involvement of actin fiber contraction in cellular alignment. This showed that the intracellular tension of actin fiber contraction balanced with intracellular tension elicited cellular alignment along the uniaxial boundary condition. Hence, the intracellular tension was strengthened by stabilizing actin filaments along the uniaxially-fixed boundary condition, and therefore, this positive tension-feedback under isometric contraction between two fixed boundaries might contribute to the enhanced intracellular tension. As illustrated in Fig. 4, our results also implied that this in vivo mechanical factor might elicit cellular alignment for osteoblasts in the bone matrix.

In this study, without applying any external mechanical stimuli, cellular alignment was achieved on the surface of the matrix using the uniaxially-fixed mechanical boundary condition exerted from the borders of the collagen matrix fixed to the PDMS frame. Interestingly, their alignment was maintained even after cells on the surface migrated into the matrix. Nonetheless, a decrease in the ratio of cellular alignment ratio at 10 – 20 μm depth inside the collagen matrix was observed (Fig. 2G,H). This might have been caused by a decrease in the stabilization of actin fibers for cells inside the collagen matrix compared with cells on the surface of the matrix. As the tight and contractile actin fibers in subcultured cells were formed along the uniaxially-fixed mechanical boundary condition on the surface of the collagen matrix, cells on the surface exerted the aligned distribution along the boundary condition. On the other hand, the actin fiber structure in cells inside the collagen matrix was relatively weakened, resulting in the less aligned cell distribution. When the aligned cells migrated inside the matrix, they encountered the randomly distributed collagen fibers and were forced to alter their original orientation. Although the rate of cellular alignment in the collagen matrix was relatively decreased compared with that on the surface of the matrix, we first reconstructed the cellular alignment in the collagen matrix using a uniaxially-fixed mechanical boundary condition. In order to fully understand the in vivo mechanical environment of unmineralized osteoid or mineralized bone matrix, measurement of stiffness and deformations in the bone matrix during the mineralization process is required. In addition, further studies will be needed to address the effect of collagen concentration on the cellular behavior so that our collagen model enables to fully reconstruct the unmineralized osteoid in the future.

After culturing the osteoblast-like cells on the collagen matrix for a long period of time, cells were observed to migrate inside the matrix and differentiate into osteocyte-like cells. In the case of fibroblast cells, these migrated and penetrated all the way underneath the gel, forming a 2D monolayer on the culture dish placed under the collagen gel^22^. Thus, the migration of osteoblast cells inside the collagen matrix is believed to have actively occurred. Regarding osteocytes differentiated from osteoblasts, a recent study reported that osteoblasts were not captured and passively embedded by surrounding osteoblasts, but they were shown to also actively penetrate the osteoid bone matrix^12^. Whereas most cells were noted to stay on the surface of the collagen (0–5 µm) after 4 days of cultivation, more than 60% of the cells actively migrated into the collagen matrix (5 µm or deeper) after 10 days. As shown in this study, the migration of cells in the matrix became deeper with longer culturing time. Taken together with the observed cellular alignment behavior, we suggest that osteocyte alignment inside the bone could be achieved by the migration of the aligned cells from the surface to the inside of the matrix while retaining their orientation as described in Fig. 4.

In addition, we also found that there was heterogeneity in the cell migratory behaviors at localized areas where the cell density was high, whereas cells in an area with relatively low cell density did not undergo migration into the matrix. Therefore, the cell condensation condition on the matrix might contribute to the heterogeneous cell infiltration. Previously, our group reported that cell condensation acquired from a 3D cell culture model triggered in vitro osteocyte differentiation of osteoblast-like cells^23,24^. Other studies also demonstrated the presence of osteocyte-like cells in the bone nodule, which has a highly localized pile of condensed cells after long-term cultivation of osteoblasts^25–27^. In our present collagen model, it is thought that condensed cells at the localized area in the collagen matrix become activated, leading to the induction of osteocyte differentiation and cellular migration. This might explain the group migration of cells in areas where the cell condensation was high.

In vivo, osteocytes differentiated from pre-osteoblasts are known to be embedded inside the hard bone matrix, resulting in a difficulty to isolate osteocytes from tissues. Many researchers have attempted to develop an in vitro osteogenic differentiation model using osteoblasts; thereby, the importance of chemical and mechanical factors for osteogenic differentiation has been revealed. Regarding the chemical factors, the use of l-ascorbic acid and β-glycerophosphate has become a gold standard for inducing osteogenesis in vitro, and was therefore utilized in our model^28–30^. On the other hand, it is thought that the mechanical properties of the matrix at which cells attach might contribute to the cell differentiation capability. Several studies recruited the collagen matrix as a three-dimensional cultivation system, resulting in the promotion of osteogenic differentiation^11,27,31,32^. Whereas those studies demonstrated the enhancement of osteogenesis after long cultivation periods of over 3 weeks, our study revealed the remarkable up-regulation in osteoblast (*Alp* and *Ocn*) and osteocyte (*Phex* and *Sost*) markers within 10 days, as depicted in Fig. 3. The relative down-regulation of *Dmp1* mRNA expression observed at day 10 compared with that at day 4 was assumed to result from the fact that the expression of DMP1 is known to be suppressed in matured osteocytes, whereas it is more apparent in osteoid- or young osteocytes^1,33^. Due to practical difficulties, we could not separate the matrix vertically to examine the gene expression changes in the migrated cells compared to the cells on the surface. For further study, it will be meaningful to examine the differential gene expression in cells that migrate to different depths of the matrix. Despite this limitation, our immunostaining results showed that the migrated cells inside the collagen matrix rendered the expression of PHEX, as actin staining represented the multiple and slender, three-dimensional cytoplasmic processes resembling osteocytes. Our results suggested that our in vitro model successfully induced osteocytogenesis with aligned cells.

In conclusion, we developed a novel in vitro collagen-based system exerting a uniaxially-fixed mechanical boundary condition. Our present study suggested that the intracellular tension of osteoblasts under uniaxially-fixed boundary conditions partially contributes to the determination of the osteocyte alignment inside the bone matrix. As there is no other model mimicking osteoblast or osteocyte alignment in vitro, our model is expected to provide great assistance to those studying osteocyte differentiation and their networks. Particularly, it has the potential to become a useful in vitro tool for osteocyte research toward revealing their mechanism of network formation or elucidating their role as a mechanosensor.

## Methods

### Fabrication of collagen matrix with fixed boundary condition

In this study, we prepared polydimethylsiloxanes (PDMS) (Silpot 184 W/C, Dow Toray, Japan) according to the manufacturer’s protocol. After setting a square-shaped (4 × 4 mm) or rectangular-shaped (4 × 12 mm) acrylonitrile butadiene styrene (ABS) plastic mold on a glass-bottom dish, the PDMS gel was poured into the dish. The PDMS with the ABS mold was then incubated in the oven at 60 °C for 12 h. After incubation, the ABS mold was gently removed to make a vacant space in the PDMS. For the all-side fixed matrix, a solution of collagen gel (Cellmatrix Type I A, Nitta-Gelatin, Japan) was used to fill the vacant space previously occupied by the square-shaped ABS mold and then incubated at 37 °C for 15 min for gelation. Figure 1A illustrates the all-side fixed matrix located inside the PDMS to exert an isotropically-fixed mechanical boundary condition. For developing the 2-side fixed matrix, the rectangular-shaped molds were utilized. After hardening the PDMS in the oven, the rectangular-shaped mold was removed from the PDMS. Two new square-shaped molds (4 × 4 mm) were then inserted into the two opposite sides of the rectangular-shaped space to create a square-shaped open space (4 × 4 mm) in the middle of the rectangular space. After filling the square-shaped open space with the collagen gel, the two pieces of square-shaped mold were removed, and the 2-side fixed matrix was consequently formed for a uniaxially-fixed mechanical boundary condition as described in Fig. 1B.

### Cell culture

The MC3T3-E1 mouse osteoblast-like cell line was utilized in this study. Cells were cultured in MEM-α (Gibco, USA) containing 10% fetal bovine serum (Gibco) and 1% antibiotic–antimycotic cocktail (Gibco) in a humidified incubator at 37 °C. Cell passage was carried out when the confluency of the cells reached up to 80–90%. The cells from passage 25 to 30 were utilized for the experiment. We subcultured the cells on the collagen matrix using a cell density of 10,000 cells/cm^2^ in an osteogenic induction medium containing 50 μg/mL ascorbic acid and 10 mM β-glycerophosphate. The osteogenic induction medium was changed every 2 days when culturing cells on the collagen matrix for 4 or 10 days.

### Immunostaining

Cells in the collagen matrix were fixed using 4% paraformaldehyde phosphate buffer solution (Wako, Japan) at room temperature for 30 min. Then, the samples were washed twice with phosphate-buffered saline (PBS) and blocked using PBS containing 3% bovine serum albumin (BSA) (Sigma, USA) at room temperature for 1 h. After removing the blocking buffer, anti-mouse PHEX (Sigma) was added to the sample and incubated at 4 °C overnight. After incubation with the primary antibody, the samples were rinsed twice with PBS. Then, the sample was permeabilized with 0.1% Triton-X (MP Biomedicals, USA) for 20 min. After rinsing twice with PBS, the samples were subjected to incubation with the secondary antibody (Anti-rabbit IgG Alexa Fluor 488, Invitrogen, USA), actin (Alexa Fluor 546 phalloidin, Invitrogen), and DAPI staining at room temperature for 1 h. Finally, the samples were rinsed twice with PBS and observed under a confocal microscope (FV3000, Olympus, Japan). Imaging data were processed using Imaris (Bitplane, UK) and ImageJ (NIH, USA).

### Measurement of cell alignment

To evaluate the cellular alignment in the samples, we utilized the alignment of nuclei. The alignment of nuclei was determined from a major axis of elliptical nuclei observed from DAPI-stained images. While the axis parallel to the mechanical boundary condition was set to 90º ($\theta$), we depicted the cell alignment from 0° to 180°. Each depicted data represents 800–1600 cells measured from 8 independent experiments to avoid sample bias. The order parameter of cell alignment, *A*, was defined as $A=-<\mathrm{cos}2\theta >$ (parallel = 1, random = 0, perpendicular = − 1).

### Real-time-PCR

After 4 or 10 d of cultivation, the collagen sample with cells was separated from the PDMS and transferred in a Precellys Lysing Tube (Bertin, France) containing 1 mL Isogen-2 (Nippon Gene, Japan). The samples were added to the lysis and homogenization system (Minilys, Bertin) for 60 s to lyse the collagen completely. After homogenizing the sample, 50 μL p-bromoanisole (Nacalai Tesque, Japan) was added to the tube. Samples were centrifuged at 12,000 × *g* for 10 min at 4 °C. Consecutively, 600 μL supernatant was transferred to a new microtube, and then the same amount of 70% ethanol was added to the tube. The mixture was transferred to a spin cartridge (PureLink RNA Mini kit; Invitrogen) and centrifuged at 12,000×*g* for 30 s. After treating with the washing buffer (PureLink RNA Mini kit; Invitrogen), RNase free water was added to dissolve the sample inside the cartridge and then collected in a new tube by centrifuging at 12,000×*g* at 4 °C for 2 min. The extracted RNA was used for cDNA synthesis using the Transcriptor Universal Reaction Buffer (Transcriptor Universal cDNA Master, Roche, Swiss) according to the manufacturer’s protocol. To perform real-time PCR (RT-PCR), we utilized the PowerUp SYBR Green Master Mix (Thermo Fisher, USA). The sequences of all PCR primers are depicted in Table 1. We examined the expression of alkaline phosphatase (*Alp*) and osteocalcin (*Ocn*), as osteoblast markers. The phosphate-regulating endopeptidase homolog X-linked (*Phex*), dentin matrix protein 1 (*Dmp1*), and sclerostin (*Sost*) were used as osteocyte markers. Beta-actin (*β-actin*) was utilized as a housekeeping gene, and all the gene expressions were normalized to that of *β-actin*, using the ΔΔCt method.

**Table 1:** Primer list.

Gene	Forward primer	Reverse primer
β-actin	GAAATCGTGCGTGACATCAAA	TGTAGTTTCATGGATGCCACAG
Alp	CTTGACTGTGGTTACTGCTGATCA	GTATCCACCGAATGTGAAAACGT
Ocn	GCTGCCCTAAAGCCAAACTCT	AGAGGACAGGGAGGATCAAGTTC
Phex	GGAAGAAAACCATTGCCAATTATT	CGCCTGCTGAGGTTTGGA
Dmp1	TGTCATTCTCCTTGTGTTCCTTTG	AGAGCTTTCAGATTCAGTATTGTGGTAT
Sost	GGAATGATGCCACAGAGGTCAT	CCCGGTTCATGGTCTGGTT

### Statistical analysis

Data were represented as the means ± standard error. For statistical analysis, a Student’s *t*-test or one-way ANOVA followed by Tukey–Kramer post-hoc test was carried out. Rayleigh’s test was performed to evaluate the cellular alignment.

## Supplementary Information

Supplementary Information 1.

Supplementary Movie S1.
