# Publisher Correction: Uniaxially fixed mechanical boundary condition elicits cellular alignment in collagen matrix with induction of osteogenesis

**DOI:** 10.1038/s41598-021-92708-9

**Published:** 2021-06-28

**Authors:** Jeonghyun Kim, Keiichi Ishikawa, Junko Sunaga, Taiji Adachi

**Affiliations:** 1 Institute for Frontier Life and Medical Sciences, Kyoto University, Kyoto, 606-8507 Japan; 2 Department of Micro Engineering, Graduate School of Engineering, Kyoto University, Kyoto, 606-8507 Japan

Correction to: *Scientific Reports* 10.1038/s41598-021-88505-z, published online 27 April 2021

The original version of this Article contained an error in Figure 1B where the collagen gel image inside the PDMS did not display correctly.

The original Figure 1 and accompanying legend appear below.

**Figure 1:**
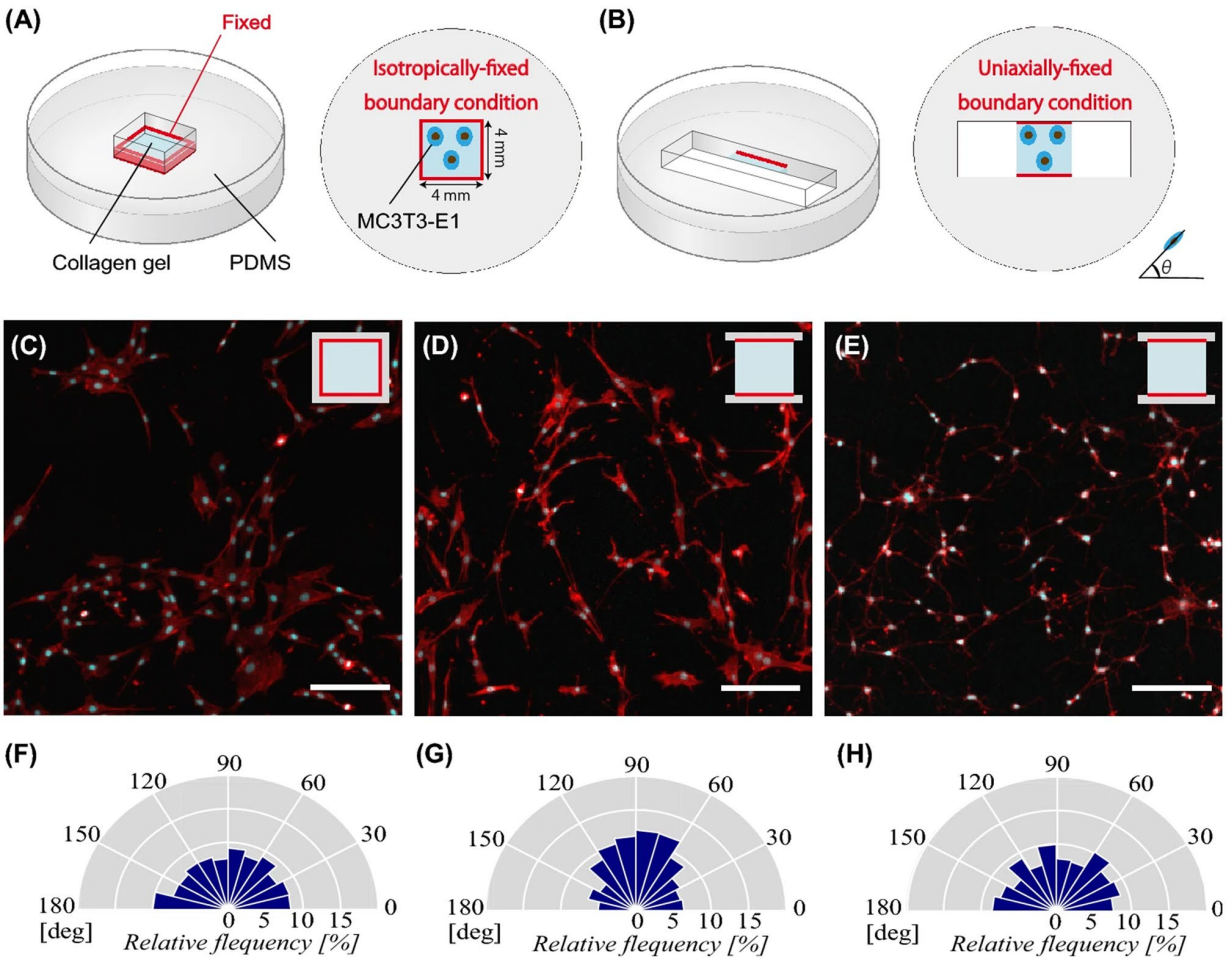
Schematic illustration of 3D collagen in vitro systems, (**A**) all-side fixed matrix and (**B**) 2-side fixed matrix, used to exert the isotropically- and uniaxially-fixed mechanical boundary condition, respectively. Images of DAPI and ACTIN staining of osteoblast-like cells cultured on (**C**) all-side fixed matrix and (**D**) 2-side fixed matrix for 1 day. (**E**) Images of DAPI and ACTIN staining of cells cultured on the 2-side fixed matrix for 1 day in the presence of blebbistatin. The ratios of cellular alignment of (**C**), (**D**), and (**E**) are depicted in (**F**), (**G**), and (**H**), respectively. Scale bars = 100 μm.

The original Article has been corrected.

