# Supplementary material for: Uniaxially fixed mechanical boundary condition elicits cellular alignment in collagen matrix with induction of osteogenesis
Source: Sci Rep. 2021 Apr 27;11:9009. doi: 10.1038/s41598-021-88505-z (PMC8079399; doi:10.1038/s41598-021-88505-z)
Supplement: Supplementary file 1 — Supplementary Information 1. [file 41598_2021_88505_MOESM1_ESM.pdf]

Supplementary Information
Uniaxially-fixed mechanical boundary condition in collagen matrix elicits cellular alignment with induction of osteogenesis

Jeonghyun Kim[a], Keiichi Ishikawa[b], Junko Sunaga[a], Taiji Adachi[a,b,*]

[a] Institute for Frontier Life and Medical Sciences, Kyoto University, Kyoto 606-8507, Japan

[b] Department of Micro Engineering, Graduate School of Engineering, Kyoto University, Kyoto 606-8507, Japan

[*]Corresponding author: Taiji Adachi, adachi@infront.kyoto-u.ac.jp Tel & Fax: +81 75 751 4853

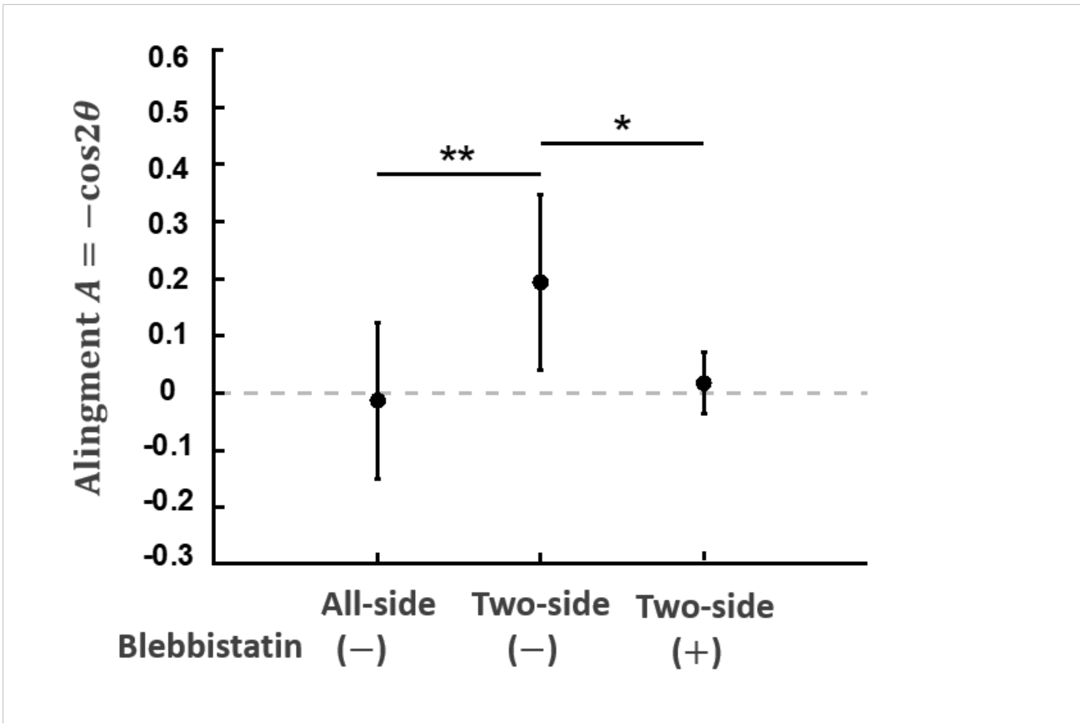

Supplementary Figure 1. Order parameter A used for quantification of the ratio of cellular alignment after 1 d incubation in the all-way fixed matrix, 2-way fixed matrix, and 2-way fixed matrix in the presence of blebbistatin. We plotted the order parameter A defined as; A=-cos2θ (perpendicular = -1, random = 0, parallel = 1). Plots represent the mean ± standard deviation (n = 8; p-value was calculated using ANOVA followed by Tukey-Kramer post-hoc test; * $p < 0.05$, ** $p < 0.01$).

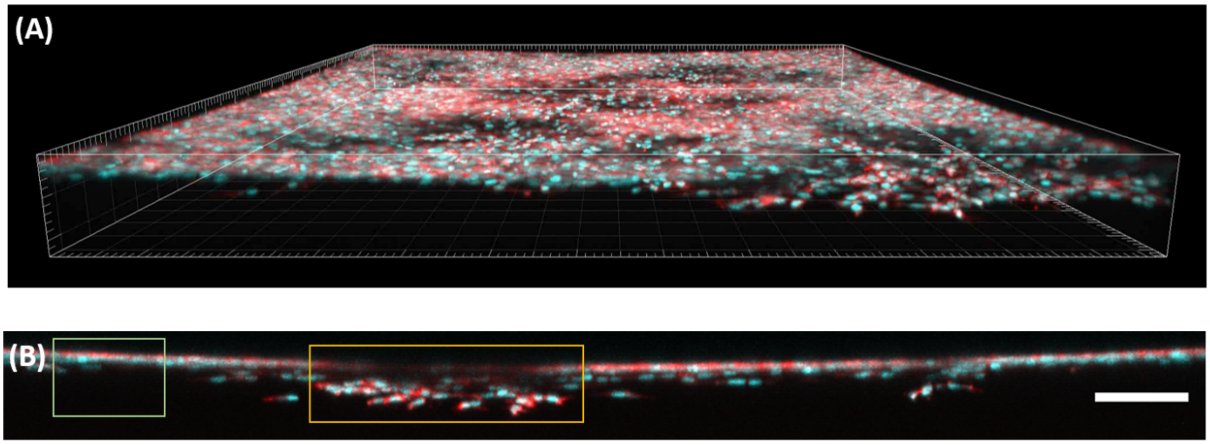

Supplementary Figure 2. Heterogeneity in cell migratory behavior in the 2-side fixed matrix. (A) Bird's-eye view and (B) vertical-section view of DAPI and ACTIN staining images of the cellular morphology for sub-cultured cells in the 2-side fixed matrix after 10 d incubation. Scale bar = 100 µm

Supplementary Movie 1. Heterogeneity in cell migratory behavior in the 2-side fixed matrix. Image of DAPI and ACTIN staining of cells sub-cultured in the 2-side fixed matrix after 10 d incubation.
